# Vam6 reduces iNKT cell function in tumor *via* modulating AMPK/mTOR pathways

**DOI:** 10.3389/fimmu.2022.1051045

**Published:** 2023-01-19

**Authors:** Shiyu Bai, Qielan Wu, Shasha Zhu, Yuwei Zhang, Xuran Chen, Miya Su, Jun Pan, Shuhang Li, Ting Yue, Linfeng Xu, Di Xie, Chenxi Tian, Dan Zhao, Xiang Li, Junjie Hou, Lu Wang, Sicheng Fu, Yanhong Xue, Amin Jiang, Dong Li, Tao Xu, Zhigang Tian, Rongbin Zhou, Huimin Zhang, Li Bai

**Affiliations:** ^1^ Department of Oncology of the First Affiliated Hospital, The CAS Key Laboratory of Innate Immunity and Chronic Disease, Division of Life Sciences and Medicine, University of Science and Technology of China, Hefei, China; ^2^ School of Basic Medical Sciences, Division of Life Sciences and Medicine, University of Science and Technology of China, Hefei, China; ^3^ Reproductive Medicine Center, Department of Obstetrics and Gynecology, The First Affiliated Hospital of Anhui Medical University, Hefei, China; ^4^ Biomedical Sciences and Health Laboratory of Anhui Province, Division of Life Sciences and Medicine, University of Science and Technology of China, Hefei, China; ^5^ Institute of Biophysics, Chinese Academy of Sciences, Beijing, China; ^6^ Institute of Molecular Medicine, Renji Hospital, School of Medicine, Shanghai Jiao Tong University, Shanghai, China; ^7^ Institute of Health and Medicine, Hefei Comprehensive National Science Center, Hefei, China; ^8^ National Synchrotron Radiation Laboratory, University of Science and Technology of China, Hefei, China

**Keywords:** Vam6, mTORC1, AMPK, Rab7a-Vam6-AMPK complex, iNKT cells

## Abstract

Activation of mTORC1 is essential for anti-tumor function of iNKT cells. The mechanisms underlying impaired mTORC1 activation in intratumoral iNKT cells remain unclear. Via generating *Vam6^+/-^* mice and using flow cytometry, image approach, and RNA sequencing, we studied the role of Vam6 in controlling mTORC1 activation and intratumoral iNKT cell functions. Here, we find that increased Vam6 expression in intratumoral iNKT cells leads to impaired mTORC1 activation and IFN-γ production. Mechanistically, Vam6 in iNKT cells is essential for Rab7a-Vam6-AMPK complex formation and thus for recruitment of AMPK to lysosome to activate AMPK, a negative regulator of mTORC1. Additionally, Vam6 relieves inhibitory effect of VDAC1 on Rab7a-Vam6-AMPK complex formation at mitochondria-lysosome contact site. Moreover, we report that lactic acid produced by tumor cells increases Vam6 expression in iNKT cells. Given the key roles of increased Vam6 in promoting AMPK activation in intratumoral iNKT cells, reducing Vam6 expression signifificantly enhances the mTORC1 activation in intratumoral iNKT cells as well as their anti-tumor effificacy. Together, we propose Vam6 as a target for iNKT cell-based immunotherapy.

## Introduction

Invariant natural killer T (iNKT) cells are innate-like T cells which express semi-invariant TCR and recognize lipid antigens presented by CD1d (1). Upon activation, iNKT cells produce both Th1 and Th2 cytokines and mediate cytotoxicity as well. These cells bridge the innate immunity and adaptive immunity (2), and are great candidates for immunotherapy against tumors, given their direct and indirect tumor killing effects and low risk of inducing cytokine storm and graft versus host disease (3). However, dysfunction of intratumoral iNKT cells has been reported to hinder their anti-tumor effects, and that is attributed to impaired activation of mTORC1 (4, 5). Previous studies have reported an essential role of mTOR pathway in functions of iNKT cells (6, 7). To date, the mechanisms underlying impaired mTORC1 activation in intratumoral iNKT cells are largely unknown.

Lysosomal proteins, lysosomal location of mTOR, and lysosome function are essential for mTORC1 activation (8). AMPK that inhibits mTORC1 activation also locates at lysosome, and its activity is controlled by lysosome related proteins (9). These findings indicate lysosome as a crucial hub for regulating mTORC1 pathway (10). Vam6 (also named Vps39), a key member of the homotypic fusion and vacuole protein sorting complex that controls tethering and fusion of lysosome with intracellular compartments (11), has been shown to regulate mTOR pathway in myoblast and senescent T cells (12, 13). However, opposite roles of Vam6 have been indicated by those two studies, and the molecular mechanisms that link Vam6 to mTOR pathway are still unknown. Whether and how Vam6 regulates mTORC1 activity and contributes to dysfunction of intratumoral iNKT cells remain to be explored.

Here, we found that Vam6 promoted AMPK activation and consequently inhibited mTORC1 activation and IFN-γ production in iNKT cells *via* forming Rab7a-Vam6-AMPK complex and recruiting AMPK to lysosome. VDAC1 interacting with Rab7a at mitochondria-lysosome contact site interfered with Rab7a-Vam6-AMPK complex formation and inhibited AMPK activation, demonstrating a negative control of this signal pathway at mitochondria-lysosome contact site. Notably, Vam6 inhibited Rab7a-VDAC1 interaction and relieved the inhibitory effect of VDAC1 on Rab7a-Vam6-AMPK complex formation. Our data propose dual roles of Vam6 in promoting AMPK activation. Moreover, we showed that lactic acid-induced increase of Vam6 expression in intratumoral iNKT cells led to increased AMPK activation, diminished mTORC1 activation, and impaired IFN-γ production. Reducing Vam6 expression, on the contrary, successfully restored function of intratumoral iNKT cells and enhanced their anti-tumor efficacy in mouse tumor models. Our data suggest that Vam6 could be a target for iNKT cell-based anti-tumor immunotherapy.

## Material and methods

### Mice

*Vam6* ^+/−^, *Vdac1* ^+/−^, and *Vdac1* ^−/−^ mice were generated using CRISPR/Cas9. *Vα14 Tg* mice were gifts from Dr. Albert Bendelac. Mice used in our experiments were 6-12 weeks old and cohoused littermates, and were on C57BL/6J background and maintained under specific pathogen-free conditions. To generate chimeric mice, bone marrow cells isolated from CD45.1 *Vam6* ^+/+^ mice and CD45.2 *Vam6^+/^* ^-^ mice were mixed at 1:1 ratio (1 × 10^6^) and then were intravenously injected into irradiated *Jα18* ^-/-^ recipient mice (10 Gy) for 7 weeks. To activate iNKT cells *in vivo*, mice were intraperitoneally injected with 2 μg α-GC for 4 hours. Animal procedures were approved by the Animal Care and Use Committee of University of Science and Technology of China, and all experiments were performed in accordance with the approved guidelines.

### Mouse tumor models

Mice were subcutaneously injected with B16F10 tumor cells (5 × 10^5^) on day 0. Tumor bearing mice were then paratumorally injected with PBS or expanded *Vam6* ^+/+^ iNKT cells (5 × 10^6^) or expanded *Vam6* ^+/-^ iNKT cells (5 × 10^6^) on day 12. Tumor size was measured by vernier caliper every 2 days and tumor volume was calculated as length × width^2^ × 0.52. In lung metastasis models, B16F10 cells (5 × 10^4^) were intravenously injected into mice on day 0. After 24 hours, PBS or expanded *Vam6^+/−^* iNKT cells (1 × 10^6^) or expanded *Vam6^+/+^* iNKT cells (1 × 10^6^) were intraperitoneally injected into these B16F10-transfered mice. On day 19 or 21, tumor-bearing mice were sacrificed for analysis of lung metastasis. To investigate the functions of lymphocytes in tumors or in lungs of metastasis models after iNKT cell transfer, *Jα18* ^-/-^ tumor-bearing mice were injected intraperitoneally with PBS or 2 μg α-GC for 4 hours, 2 days post transfer of expanded *Vam6^+/+^* or *Vam6* ^+/-^ iNKT cells. Tumor-bearing animals were euthanized if they exhibited signs of distress or the tumor reached a diameter of 1.60 cm.

### Cell stimulation and expansion

To measure the cytokine production in supernatants, iNKT cells from livers of *Vam6* ^+/+^ or *Vam6^+/-^* mice were isolated as TCRβ^+^ CD1d-PBS57 tetramer^+^ cells by a BD influx cell sorter (BD FACSAriaIII, Franklin Lakes, US). Cells were stimulated with or without plate-coated 2 μg/mL CD1d-PBS57 tetramer for 48 hours, and cytokines in supernatants were measured using cytometric bead array kit (BD, 558296 and BD, 558298). To measure the intracellular cytokines, activation of AMPK and mTORC1, cell proliferation and apoptosis, magnetic beads (Miltenyi Biotec, Bergisch Gladbach, Germany)-enriched splenic iNKT cells were stimulated with plate-coated 4 μg/mL anti-CD3 plus 4 μg/mL anti-CD28 antibodies for 4 hours, or with 2 μg/mL CD1d-PBS57 tetramer for 18 hours, or with 1 μg/mL anti-CD3 plus 1 μg/mL anti-CD28 antibodies for 18 hours *in vitro*. To inhibit or activate AMPK, 10 nM Compound C (Selleck, Sylvanfield, US) or 200 μM AICAR (Sigma, St. Louis, US) was added to cells in the last 30 minutes. To inhibit mTORC1, 100 μM rapamycin (Sigma, St. Louis, US) was added to cells in the last 30 minutes. To expand iNKT cells *in vitro*, spleen cells from *Vα14 Tg/Vam6^+/−^* mice or *Vα14 Tg/Vam6^+/+^* mice were stimulated with 100 ng/mL α-GC for 3 days in the presence of 200 IU/mL IL-2, and then were cultured for another 4 to 7 days in the presence of IL-2, the purity of iNKT cells was about 80%.

### Sequence alignment and analysis

cDNA library was sequenced using the Illumina sequencing platform (NovaSeq6000). The size of the library was ~300 bp, and both ends of the library were sequenced to a length of 100 bp. The raw reads were cleaned by removing adaptor sequences, short sequences (length < 35 bp), low-quality bases (quality < 20). and ambiguous sequences (i.e., reads with more than two unknown bases ‘N’). We used Hisat2 (version:2.0.4) to map the cleaned RNA-seq reads to the mouse mm10 genome, with two mismatches, two gaps, and one multihit allowed. After genome mapping, Stringtie (version:1.3.0) was used to quantify gene expression. The gene expression value was normalized by FPKM and adjusted by a geometric algorithm. The accession number for the RNA-seq data is the National Center for Biotechnology Information Sequence Read Archive (NCBI SRA): SUB12104972.

### Cell transfection

To knock down *Vam6* in NIH-3T3 cells, we generated lentiviruses carrying the sh*Vam6* sequences (Sigma, St. Louis, US). The lentivirus-containing media were collected and added to NIH-3T3 cells for 48 hours to knock down *Vam6* in the presence of 6 μg/mL Polybrene (Sigma-Aldrich, St. Louis, US). To restore expression of Vam6 or its truncated mutants in *Vam6* knockdown NIH-3T3 cells, lentiviruses carrying m*Cherry-Vam6*, m*Cherry-*ΔCT, m*Cherry-*ΔCLH, m*Cherry-*ΔCNH, and *mCheery* as control were packaged in 293T cells and were used to transduce target genes, respectively.

### PLA

After surface staining of CD1d-PBS57 tetramer, enriched iNKT cells were fixed, permeabilized, and blocked, followed by intracellular staining with primary antibodies against Tom20 (CST, Danvers, US) and LAMP2 (Thermo Fisher, Waltham, US), against VDAC1 (Abcam, Cambridge, UK) and Rab7a (Sigma, St. Louis, US), against AMPKγ (Invitrogen, Carlsbad, US) and Rab7a (Sigma, St. Louis, US), against Vam6 (Thermo Fisher, Waltham, US) and Rab7a (Sigma, St. Louis, US), and against Vam6 (Thermo Fisher, Waltham, US) and AMPKγ3 (Invitrogen, Carlsbad, US), respectively. As negative controls, cells were stained with rabbit IgG (Invitrogen, Carlsbad, US) and mouse IgG2b (Invitrogen, Carlsbad, US), or stained with rabbit IgG (Invitrogen, Carlsbad, US) and mouse IgG1 (biolegend, San Diego, US). After washing, cells were stained with PLA detection reagents according to the manufacturer’s instructions (Duolink, Sigma). For colocalization analysis, cells were incubated with 500 nM Mitotracker Deep Red (Thermo Fisher, Waltham, US) for 30 minutes at 37 °C before surface staining, or stained with antibody against LAMP2 (eBioscience, San Diego, US) after PLA staining. PLA puncta in CD1d-PBS57 tetramer^+^ cells were detected by confocal microscope (ZEISS LSM980, Oberkochen, Germany) with a 40/63/100 × oil objective or ImageStreamX imaging flow cytometry (Merck Millipore, Burlington, US) with 40 × magnification. Images were analyzed with ImageJ software (Fiji) or with IDEAS software.

### Antibodies and flow cytometry

After blocking with anti-CD16/32 (Biolegend, San Diego, US), cells were stained with antibodies against surface molecules. For intracellular staining, cells were fixed and permeabilized with foxp3 staining buffer kit (eBioscience, San Diego, US) after surface staining, followed by staining with antibodies against intracellular molecules. Fluorophore-conjugated or unconjugated antibodies included anti-TCRβ (Biolegend, San Diego, US), anti-PLZF (Biolegend, San Diego, US), anti-RORγt (Biolegend, San Diego, US), anti-CD24 (Biolegend, San Diego, US), anti-Granzyme B (Biolegend, San Diego, US), anti-IFN-γ (Biolegend, San Diego, US), anti-IL-4 (Biolegend, San Diego, US), anti-CD45.1 (Biolegend, San Diego, US), anti-CD45.2 (Biolegend, San Diego, US), anti-B220 (Biolegend, San Diego, US), anti-Bcl2 (Biolegend, San Diego, US), anti-p-S6^S235/236^ (CST, Danvers, US), anti-AMPKα (Abcam, Cambridge, UK), anti-p-AMPKα (Invitrogen, Carlsbad, US), anti-Vam6 (Thermo Fisher, Waltham, US), anti-VDAC1 (Abcam, Cambridge, UK), anti-Rab7a (NewEast, Kelayres, US), anti-LAMP2 (Invitrogen, Carlsbad, US), and anti-Ki67 (BD Pharmingen, San Diego, US). Apoptosis was measured using Annexin V apoptosis detection kit with PI (Biolegend, San Diego, US). CD1d-PBS57 tetramer was provided by the NIH Tetramer Core Facility. Secondary antibodies included donkey-anti-rabbit IgG-PE (Biolegend, San Diego, US) and goat-anti-rabbit IgG-FITC (Jackson Immunoresearch). Isotype controls included BV510 rat IgG1 (Biolegend, 400435), BV421 rat IgG1 (Biolegend, San Diego, US), PE mouse IgG1 (Biolegend, San Diego, US), rabbit IgG (Invitrogen, Carlsbad, US), Alexa Fluor 488 rabbit IgG (CST, Danvers, US), and APC rat IgG2a (eBioscience, San Diego, US). Samples were acquired with a BD FACSVerse flow cytometry, and data were analyzed with FlowJo software (TreeStar).

### Co-immunoprecipitation and immunoblotting

Antibodies were incubated with Dynabeads protein G (Invitrogen, Carlsbad, US) at 4°C for at least 30 minutes. Cells were lysed in NP-40 buffer (Beyotime, Shanghai, China) supplemented with protease inhibitor cocktail (Thermo Fisher, Waltham, US). Cell lysate was incubated with antibody-coated beads at 4 °C overnight. Antibodies used for immunoblotting and co-immunoprecipitation included anti-Vam6 (Thermo Fisher, Waltham, US), anti-VDAC1 (Abcam, Cambridge, UK), anti-Rab7a (NewEast, Kelayres, US), anti-AMPKγ (Invitrogen, Carlsbad, US), anti-mCherry (Invitrogen, Carlsbad, US), anti-β-actin (Proteintech, Chicago, US), and rabbit IgG isotype ctrl (Invitrogen, Carlsbad, US). HRP-conjugated secondary antibodies included mouse-anti-rabbit IgG light chain (CST, Danvers, US), goat-anti-mouse IgG (H+L) (Proteintech, Chicago, US), and goat-anti-rabbit IgG (H+L) (Proteintech, Chicago, US). Antibodies and isotype controls used in co-immunoprecipitation experiments were at 1 μg/mL, and antibodies for immunoblotting were used at 1:1000 dilution unless other described.

### Statistical analysis

Statistical analyses were performed with two-tailed unpaired Student’s t-test, Mann-Whitney test, Wilcoxon matched-pairs signed rank test, two-way ANOVA, Pearson correlation, and log-rank (Mantel-Cox) test, using GraphPad Prism software. Colocalization analyses were performed with ImageJ software (Fiji). *P<0.05, **P<0.01, ***P<0.001, and ****P<0.0001 were considered statistically significant. ns, not significant.

## Results

### Vam6 upregulation inhibits function of intratumoral iNKT cells

**Figure 1:**
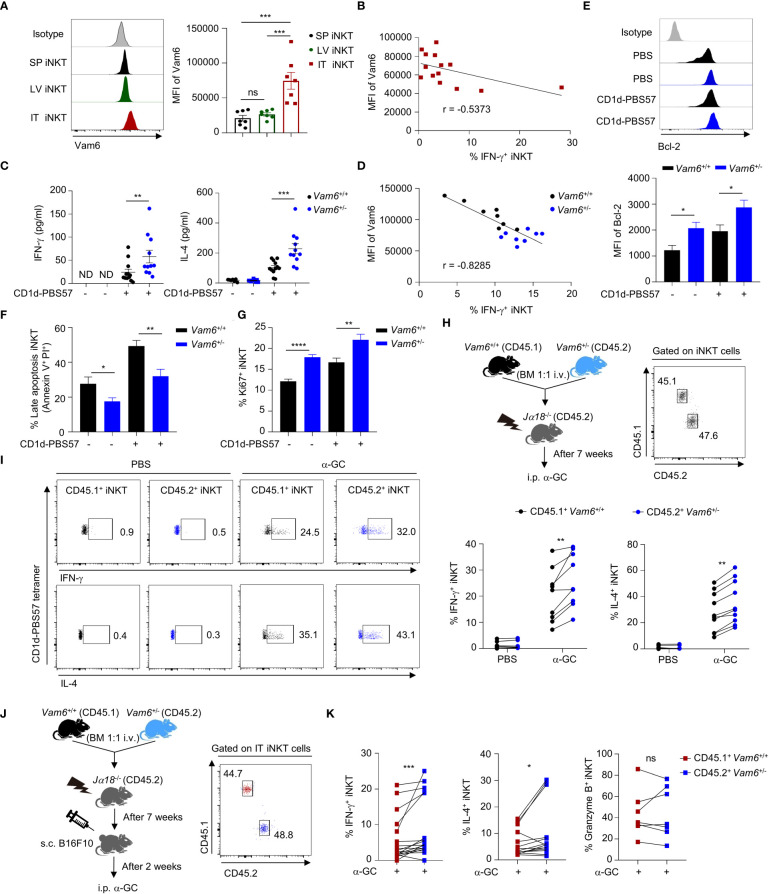
Increased Vam6 in intratumoral iNKT cells leads to impaired IFN-γ production. **(A)** Vam6 expression in splenic (SP), hepatic (LV), and intratumoral (IT) iNKT cells from B16F10 tumor-bearing mice, 4 hours post α-GC injection. n = 7 mice for each group. **(B)** Correlation between Vam6 and IFN-γ production in IT iNKT cells from tumor-bearing mice, 4 hours post α-GC injection. n = 14 mice. **(C)** Supernatant IFN-γ and IL-4 produced by sorted hepatic iNKT cells from *Vam6* ^+/+^ and *Vam6* ^+/-^ mice, stimulated with or without plate-coated CD1d-PBS57 tetramer for 48 hours. n = 11-12 mice for each group. ND, not detected. **(D)** Correlation between Vam6 and IFN-γ production in *Vam6* ^+/+^ and *Vam6* ^+/-^ SP iNKT cells, stimulated with CD1d-PBS57 tetramer *in vitro*. n = 16 mice. **(E-G)** Bcl-2 expression **(E)**, frequencies of apoptotic cells **(F)**, and frequencies of Ki67^+^ cells **(G)** in *Vam6* ^+/+^ and *Vam6* ^+/-^ iNKT cells stimulated with or without CD1d-PBS57 tetramer for 18 hours. n = 9-12 samples for each group. **(H)** Experimental procedure for **(I)**. **(I)** IFN-γ and IL-4 production in CD45.1^+^ *Vam6* ^+/+^ iNKT cells and CD45.2^+^ *Vam6* ^+/-^ iNKT cells from spleens of chimeric mice, 4 hours after α-GC or PBS injection. n = 7-10 mice for each group. **(J)** Experimental procedure for **(K)**. **(K)** IFN-γ, IL-4, and Granzyme B production in CD45.1^+^ *Vam6* ^+/+^ iNKT cells and CD45.2^+^ *Vam6* ^+/-^ iNKT cells in B16F10 tumors from chimeric mice, 4 hours post α-GC injection. n = 7-17 mice. Data are shown as mean ± SEM **(A, C, E**-**G)** and pooled from two **(A, B, D)**, three **(C, E**-**G)**, or four **(I, K)** independent experiments. Data were analyzed by two-tailed Mann-Whitney tests **(A, C, E**-**G)**, two-tailed Wilcoxon matched-pairs signed rank tests **(I, K)**, and Pearson correlation **(B**, **D)**. *P < 0.05, **P < 0.01, ***P < 0.001, ****P < 0.0001. ns, not significant. See also **Supplementary Figure 1**.

Previous study has reported dysfunction of intratumoral iNKT cells (4), but the underlying mechanisms remain unclear. We found that intratumoral iNKT cells expressed higher level of Vam6 than iNKT cells from normal tissues, such as livers and spleens (**Figure 1A**), and Vam6 expression in intratumoral iNKT cells was negatively correlated with their IFN-γ production in response to α-GC injection (**Figure 1B**). Next, we investigated whether the upregulated Vam6 influenced iNKT cell function in tumors. Since *Vam6^-/-^* homozygous mice were embryonically lethal (12), we generated *Vam6^+/-^* mice and confirmed reduction of Vam6 expression in these animals using splenic *Vam6^+/-^* T cells (**Supplementary Figure 1A**). Reducing Vam6 expression in *Vam6^+/-^* mice did not influence the frequencies of iNKT cells in thymuses, spleens, and livers (**Supplementary Figures 1B, C**). The absolute numbers of thymic and splenic iNKT cells were normal in *Vam6^+/-^* mice, despite the slightly elevated numbers of hepatic iNKT cells (**Supplementary Figure 1C**). Moreover, the proportions of thymic iNKT1/2/17 subsets were not influenced by reducing Vam6 expression (**Supplementary Figure 1D**). These results demonstrate normal iNKT cell development in *Vam6^+/-^* mice. To study the influence of reduced Vam6 expression on iNKT cell function, we sorted iNKT cells from livers of *Vam6^+/-^* mice and *Vam6^+/+^* mice, respectively, and activated them with CD1d-PBS57 tetramer *in vitro*. Upon activation, *Vam6^+/-^* iNKT cells produced more IFN-γ and IL-4 in supernatants than *Vam6^+/+^* iNKT cells did (**Figure 1C**). Again, the level of CD1d-PBS57 tetramer-induced intracellular IFN-γ was negatively correlated with the level of Vam6 in *Vam6^+/-^* iNKT cells and *Vam6^+/+^* iNKT cells (**Figure 1D**). Additionally, *Vam6^+/-^* iNKT cells expressed higher level of anti-apoptosis protein Bcl-2 (**Figure 1E**), exhibited lower apoptosis (**Figure 1F**) but higher proliferation (**Figure 1G**). To confirm the role of Vam6 in controlling iNKT cell function *in vivo*, we measured the intracellular cytokines, 4 hours after injecting lipid antigen α-galactosylceramide (α-GC) into lethally irradiated iNKT-deficient *Jα18^-/-^* mice that received CD45.2*^+^ Vam6^+/-^* bone marrows mixed with CD45.1*^+^ Vam6^+/+^* bone marrows at a ratio of 1:1 (**Figure 1H**). Consistently, reduced expression of Vam6 did not influence the development of iNKT cells in these chimeric mice, and CD45.2*^+^ Vam6^+/-^* iNKT cells exhibited higher levels of IFN-γ and IL-4 production than CD45.1*^+^ Vam6^+/+^* iNKT cells did (**Figures 1H, I**). To prove that increased Vam6 led to impaired iNKT cell functions in tumors, we generated B16F10 tumor-bearing *Jα18^-/-^* chimeric mice, in which we found that those intratumoral iNKT cells with reduced Vam6 expression produced more IFN-γ and IL-4 in response to α-GC injection, despite their normal granzyme B production (**Figures 1J, K**). Together, our data demonstrate that the upregulation of Vam6 leads to iNKT cell dysfunction in tumors.

### Vam6 inhibits iNKT cell functions *via* modulating AMPK/mTOR pathways

**Figure 2:**
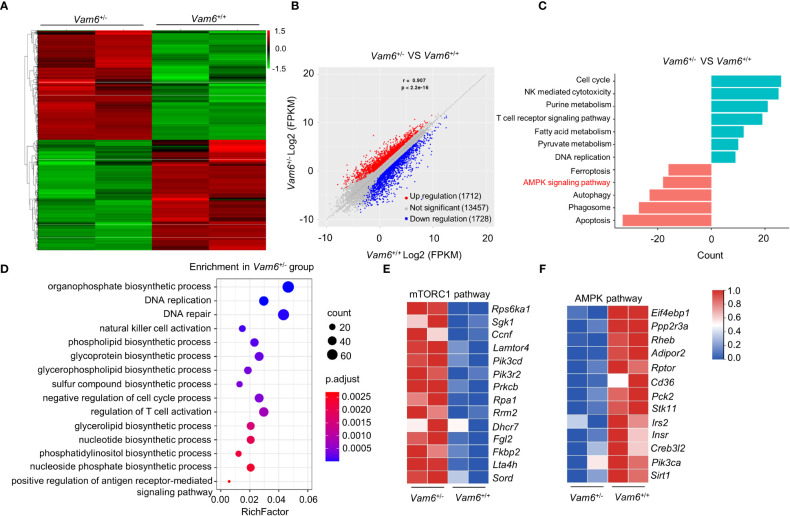
mRNA-seq data reveal the altered AMPK/mTORC1 signaling in *Vam6* ^+/-^ iNKT cells. **(A)** Heatmap displaying 3440 significantly dysregulated genes in *Vam6* ^+/-^ iNKT cells in comparison with *Vam6* ^+/+^ iNKT cells after stimulation with CD1d-PBS57 tetramer for 4 hours. The significance threshold is fold change ≥ 2 and FDR ≤ 0.05. **(B)** Correlation plot displaying expression of genes in *Vam6* ^+/-^ iNKT cells and in *Vam6* ^+/+^ iNKT cells. **(C)** KEGG pathway analysis showing pathways downregulated and upregulated in *Vam6* ^+/-^ iNKT cells. Bars represent the number of differentially expressed genes contributing to each gene set. **(D)** GO term analysis showing pathways upregulated in *Vam6* ^+/-^ iNKT cells. **(E, F)** Heatmaps showing expression of genes involved in mTORC1 pathway **(E)** and AMPK pathway **(F)** in *Vam6* ^+/-^ iNKT cells and in *Vam6* ^+/+^ iNKT cells.

To study the role of Vam6 in controlling iNKT cell function, we performed RNA-seq experiments and found that reducing Vam6 expression altered gene expression profile in iNKT cells (**Figures 2A, B**). 1712 genes were upregulated whereas 1728 genes were downregulated in *Vam6^+/-^* iNKT cells. KEGG (Kyoto Encyclopedia of Genes and Genomespathway) analysis and GO (Gene Ontology) term analysis showed that the upregulated genes were enriched in cell cycle, cell activation, and metabolic processes, whereas the downregulated genes were enriched in cell death, autophagy, and AMPK pathway. The gene profile of *Vam6^+/-^* iNKT cells was in line with their increased proliferation, survival, and function (**Figures 2C, D**). It is well-known that cell growth and metabolic processes are promoted by mTORC1 (14) whereas the autophagy is inhibited by mTORC1 (15). Additionally, AMPK is a negative regulator of mTORC1 (16, 17). Therefore, our gene enrichment analysis implied alterations in AMPK/mTOR pathways in *Vam6^+/-^* iNKT cells. Indeed, we found increased expression of genes related to mTORC1 pathway (**Figure 2E**) and decreased expression of genes related to AMPK pathway (**Figure 2F**) in these iNKT cells with reduced Vam6 expression.

**Figure 3:**
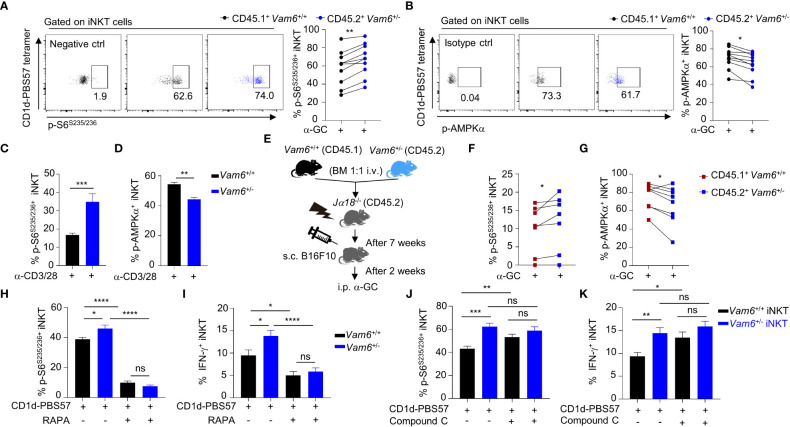
Reduced Vam6 expression promotes mTORC1 activation and IFNγ production *via* suppressing AMPK activation. **(A**, **B)** Phosphorylation of S6^S235/S236^ **(A)** and phosphorylation of AMPKα **(B)** in CD45.1^+^ *Vam6* ^+/+^ and CD45.2^+^ *Vam6* ^+/-^ splenic iNKT cells in mixed bone marrow chimeras, after α-GC injection, as described in Figure 1H. n = 10 mice for each group. **(C**, **D)** Phosphorylation of S6^S235/S236^ **(C)** and phosphorylation of AMPKα **(D)** in anti-CD3 plus anti-CD28-stimulated splenic iNKT cells from *Vam6* ^+/+^ and *Vam6* ^+/-^ mice. n = 6-9 replicates for each group. **(E-G)** Experimental procedure **(E)**, phosphorylation of S6^S235/S236^ **(F)**, and phosphorylation of AMPKα **(G)** in CD45.1^+^ *Vam6* ^+/+^ and CD45.2^+^ *Vam6* ^+/-^ intratumoral iNKT cells in chimeric mice, after α-GC injection. n = 7-8 mice for each group. **(H**, **I)** Phosphorylation of S6^S235/S236^ **(H)** and IFN-γ production **(I)** in CD1d-PBS57 tetramer-stimulated splenic iNKT cells from *Vam6* ^+/+^ and *Vam6* ^+/-^ mice, in the presence or absence of rapamycin. n = 12 replicates for each group. **(J**, **K)** Phosphorylation of S6^S235/S236^ **(J)** and IFN-γ production **(K)** in CD1d-PBS57 tetramer-stimulated splenic iNKT cells from *Vam6* ^+/+^ and *Vam6* ^+/-^ mice, in the presence or absence of Compound C. n = 9 replicates for each group. Data are shown as mean ± SEM **(C**, **D**, **H**-**K)** and pooled from two **(D**, **F**, **G)**, three **(C, H**-**K)**, or four **(A**, **B)** independent experiments. Data were analyzed by two-tailed Mann-Whitney tests **(C**-**D** and **H**-**K)** and two-tailed Wilcoxon matched-pairs signed rank tests **(A**, **B**, **F**, **G)**. *P < 0.05, **P < 0.01, ***P < 0.001, ****P < 0.0001. ns, not significant.

Next, we measured activation of mTORC1 and AMPK at protein levels, as indicated by the phosphorylation of S6^S235/236^ and phosphorylation of AMPKα, respectively. CD45.2*^+^ Vam6^+/-^* iNKT cells in *Jα18^-/-^* chimeric mice exhibited elevated mTORC1 activation (**Figure 3A**) and diminished AMPK activation (**Figure 3B**) after α-GC injection, in comparison with CD45.1*^+^ Vam6^+/+^* iNKT cells. Consistently, enhanced mTORC1 activation (**Figure 3C**) and diminished AMPK activation (**Figure 3D**) were detected in *Vam6^+/-^* iNKT cells when they were stimulated with anti-CD3 plus anti-CD28 *in vitro*. In B16F10 tumor-bearing *Jα18^-/-^* chimeric mice (**Figure 3E**), CD45.2*^+^Vam6^+/-^* iNKT cells displayed higher level of mTORC1 activation (**Figure 3F**) and lower level of AMPK activation (**Figure 3G**) than CD45.1*^+^ Vam6^+/+^* iNKT cells after α-GC injection. Activation of mTORC1 has been previously reported to promote cytokine production in iNKT cells (5). Notably, reducing Vam6 expression failed to enhance CD1d-PBS57 tetramer-induced IFN-γ production in iNKT cells treated with mTORC1 inhibitor rapamycin, further confirming that reduced Vam6 increased iNKT cell function *via* promoting mTORC1 activation (**Figures 3H, I**). Given the inhibitory effect of AMPK on mTORC1 activation (17), our results indicate that Vam6 inhibits mTORC1 activation *via* promoting AMPK activation. We found that inhibiting AMPK activation *via* Compound C increased mTORC1 activation and IFN-γ production in CD1d-PBS57 tetramer-stimulated *Vam6^+/+^* iNKT cells but not in *Vam6^+/-^* iNKT cells (**Figures 3J, K**). Together, our results demonstrate that Vam6 in iNKT cells inhibits mTORC1 activation and IFN-γ production *via* promoting AMPK activation.

### Vam6 promotes activation of AMPK *via* recruiting AMPK to lysosome

**Figure 4:**
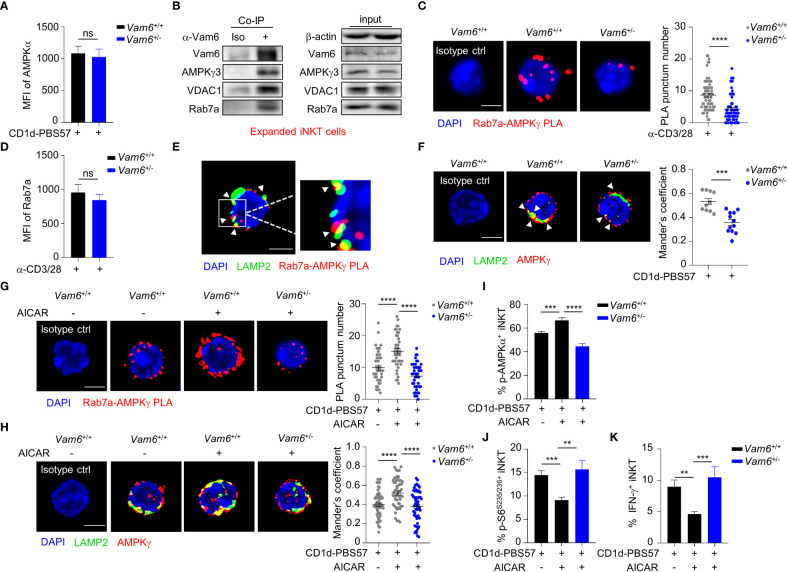
Vam6 promotes Rab7a-AMPKγ interaction and recruits AMPK to lysosome. **(A)** Protein level of AMPKα in *Vam6* ^+/+^ and *Vam6* ^+/-^ iNKT cells stimulated with CD1d-PBS57 tetramer. n = 12 replicates for each group. **(B)** Co-immunoprecipitation of Vam6 with AMPKγ3, VDAC1, and Rab7a in expanded iNKT cells. **(C)** Representative Rab7a-AMPKγ PLA images (left) and PLA punctum number per cell (right) in *Vam6* ^+/+^ and *Vam6* ^+/-^ iNKT cells stimulated with anti-CD3 plus anti-CD28 for 4 hours. n = 60-65 cells for each group. Scale bar, 3 µm. **(D)** Protein level of Rab7a in *Vam6* ^+/+^ and *Vam6* ^+/-^ iNKT cells stimulated with anti-CD3 plus anti-CD28 for 4 hours. n = 9 replicates for each group. **(E)** Representative confocal image showing colocalization of Rab7a-AMPKγ PLA puncta and lysosomal LAMP2 in iNKT cells stimulated with CD1d-PBS57 tetramer for 4 hours. Scale bar, 3 µm. **(F)** Colocalization of AMPKγ and LAMP2 in *Vam6* ^+/+^ and *Vam6* ^+/-^ iNKT cells stimulated with CD1d-PBS57 tetramer for 4 hours. Scale bar, 3 µm. **(G**, **H)** Rab7a-AMPKγ PLA puncta **(G)** and colocalization of AMPKγ and LAMP2 **(H)** in *Vam6* ^+/+^ and *Vam6* ^+/-^ iNKT cells stimulated with CD1d-PBS57 tetramer for 4 hours in the presence or absence of AICAR. n = 41-55 cells for each group. Scale bar, 3 µm. **(I-K)** Phosphorylation of AMPKα **(I)**, phosphorylation of S6^S235/S236^ **(J)**, and IFN-γ production **(K)** in *Vam6* ^+/+^ and *Vam6* ^+/-^ iNKT cells stimulated with CD1d-PBS57 tetramer in the presence or absence of AICAR. n = 9-12 replicates for each group. Data are shown as mean ± SEM **(A**, **C**, **D**, **F**-**K)** and pooled from three **(A**, **C**, **D**, **G**, **J**, **K)** or four **(H–I)** independent experiments. Data were analyzed by two-tailed unpaired Student’s t test **(C**, **F**–**H)** and two-tailed Mann-Whitney tests **(A**, **D**, **I**–**K)**. **P < 0.01, ***P < 0.001, ****P < 0.0001. ns, not significant. See also **Supplementary Figures 2, 3**.

Despite the reduced phosphorylation of AMPKα, *Vam6^+/-^* iNKT cells expressed similar level of AMPKα protein as *Vam6^+/+^* cells did (**Figure 4A**). Then, we investigated whether Vam6 promoted AMPKα phosphorylation *via* protein interactions. We performed co-immunoprecipitation experiments with expanded iNKT cells (**Figure 4B** and **Supplementary Figure 2A**), in which reducing Vam6 expression (**Supplementary Figure 2B**) increased IFN-γ production (**Supplementary Figures 2C, D**) and S6^S235/236^ phosphorylation (**Supplementary Figure 2E**) whereas diminished AMPKα phosphorylation (**Supplementary Figure 2F**), as it did in fresh iNKT cells. We found that AMPKγ3, Rab7a, and VDAC1 were co-immunoprecipitated with Vam6 (**Figure 4B**), indicating interactions between these proteins. Rab7a is a marker protein for lysosome (18). Recruiting AMPK to lysosome has been reported to be essential for AMPK activation (9). Next, we investigated whether Vam6 regulated recruitment of AMPK to lysosome *via* protein interactions. Using *in situ* proximity ligation assay (PLA) approach, we showed that AMPKγ interacted with Rab7a in activated iNKT cells, and reducing Vam6 expression impaired the Rab7a-AMPKγ interaction, as indicated by reduced Rab7a-AMPKγ PLA puncta numbers (**Figure 4C**). Additionally, we proved that reducing Vam6 expression did not influence the protein level of Rab7a (**Figure 4D**). In line with the lysosomal location of Rab7a, the Rab7a-AMPKγ complex was colocalized with lysosome marker LAMP2 (**Figure 4E**), indicating lysosomal location of Rab7a-AMPKγ interaction. In agreement with the reduced Rab7a-AMPKγ interaction in activated *Vam6^+/-^* iNKT cells, these cells had less amount of AMPK on lysosome in comparison with activated *Vam6^+/+^* iNKT cells (**Figure 4F**). These data demonstrate that Vam6 promotes Rab7a-AMPKγ interaction and recruits AMPK to lysosome. Notably, when AMPK in CD1d-PBS57 tetramer-stimulated iNKT cells was activated by AICAR, an AMP analog, the Rab7a-AMPKγ PLA puncta numbers (**Figure 4G**), the colocalization coefficient of AMPKγ and LAMP2 (**Figure 4H**), and the AMPKα phosphorylation (**Figure 4I**) was all increased in a Vam6 dependent manner. As a consequence, the inhibitory effects of AICAR on mTORC1 activation (**Figure 4J**) and on IFN-γ production (**Figure 4K**) depended on Vam6 as well. Together, these data demonstrate an essential role of Vam6 in recruiting AMPK to lysosome and activating AMPK, and exclude the possibility of regulating AMPK activation *via* influencing AMP production.

To understand how Vam6 regulated Rab7a-AMPKγ interaction, we generated various truncated Vam6 based on previous study (19), including the C terminal-deleted ΔCT, the middle fragment CLH-deleted ΔCLH, and the N terminal-deleted ΔCNH (**Supplementary Figure 3A**), and showed that Rab7a bound wide-type Vam6 as well as ΔCT but not ΔCNH or ΔCLH, and AMPKγ3 only bound wide-type Vam6 (**Supplementary Figure 3B**). These results suggest that Rab7a binds Vam6 in a N terminal and CLH fragment dependent manner, and AMPK is dispensable for their interaction. Although ΔCT interacted with Rab7a, it failed to recruit AMPK, suggesting a key role of Vam6 C terminal in forming Rab7a-Vam6-AMPK complex. We could not exclude the possibility that AMPK might not directly bind Rab7a but indirectly interacts with Rab7a through Vam6.

### Vam6 relieves inhibitory effect of VDAC1 on Rab7a-Vam6-AMPK complex formation

**Figure 5:**
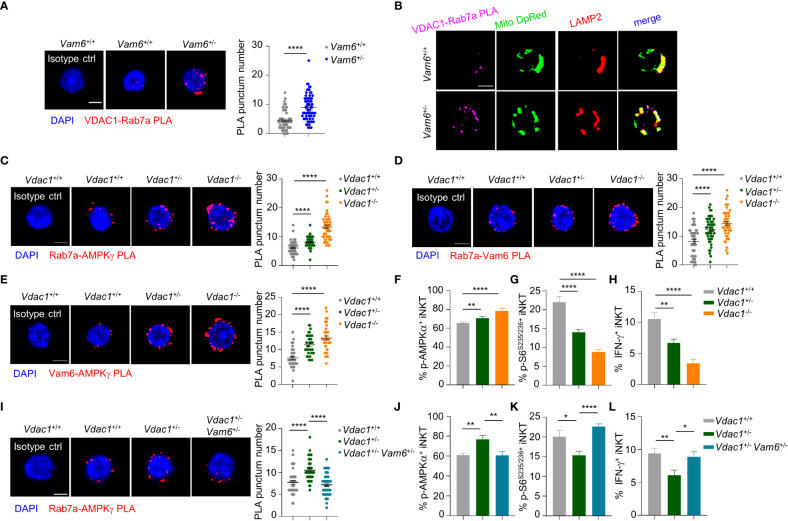
VDAC1 at mitochondria*-*lysosome contact site interferes with Vam6- Rab7a-AMPKγ complex formation and AMPK activation. **(A)** VDAC1-Rab7a PLA puncta in *Vam6* ^+/+^ and *Vam6* ^+/-^ iNKT cells stimulated with anti-CD3 plus anti-CD28 for 4 hours. n = 62-66 cells for each group. Scale bar, 3 μm. **(B)** Representative confocal images showing colocalization between VDAC1-Rab7a PLA puncta, Mitotracker deep red, and lysosomal LAMP2 in *Vam6* ^+/+^ and *Vam6* ^+/-^ iNKT cells stimulated with CD1d-PBS57 tetramer for 4 hours. Scale bar, 3 μm. **(C–E)** Rab7a-AMPKγ PLA puncta **(C**, n = 46-56 cells for each group**)**, Rab7a-Vam6 PLA puncta **(D**, n = 45-49 cells for each group**)**, and Vam6-AMPKγ PLA puncta **(E**, n = 33-37 cells for each group**)** in *Vdac1* ^+/+^, *Vdac1* ^+/-^, and *Vdac1* ^-/-^ iNKT cells stimulated with CD1d-PBS57 tetramer for 4 hours. Scale bar, 3 μm. **(F–H)** Phosphorylation of AMPKα **(F)**, phosphorylation of S6^S235/S236^ **(G)**, and IFN-γ production **(H)** in *Vdac1* ^+/+^, *Vdac1* ^+/-^, and *Vdac1* ^-/-^ iNKT cells stimulated with CD1d-PBS57 tetramer for 18 hours. n = 12-16 replicates for each group. **(I)** Rab7a-AMPKγ PLA puncta in *Vdac1* ^+/+^, *Vdac1* ^+/-^, and *Vdac1* ^+/-^ *Vam6* ^+/-^ iNKT cells stimulated with CD1d-PBS57 tetramer for 4 hours. n = 44-51 cells for each group. Scale bar, 3 μm. **(J–L)** Phosphorylation of AMPKα **(J)**, phosphorylation of S6^S235/S236^ **(K)**, and IFN-γ production **(L)** in *Vdac1* ^+/+^, *Vdac1* ^+/-^, and *Vdac1* ^+/-^ *Vam6* ^+/-^ iNKT cells stimulated with CD1d-PBS57 tetramer for 18 hours. n = 12-16 replicates for each group. Data are shown as mean ± SEM **(A**, **C**–**L)** and pooled from two **(E)**, three **(A**, **C**, **D**, **G–J)**, or four **(F**, **K, L)** independent experiments. Data were analyzed by two-tailed unpaired Student’s t test **(A**, **C**–**E**, **I)** and two-tailed Mann-Whitney tests **(F**–**H, J–L)**. *P < 0.05, **P < 0.01, ****P < 0.0001. See also **Supplementary Figures 4–6**.

Among the proteins co-immunoprecipitated with Vam6, we detected a mitochondrial protein VDAC1 (**Figure 4B**). In activated iNKT cells, reducing Vam6 expression promoted VDAC1-Rab7a interaction, as indicated by increased puncta numbers of VDAC1-Rab7a PLA (**Figure 5A**). Given the fact that VDAC1 and Rab7a are mitochondrial protein and lysosomal protein, respectively (20, 21), the VDAC1-Rab7a interaction might occur at the contact site between lysosome and mitochondria. Indeed, we found that VDAC1-Rab7a interaction sites were colocalized well with mitochondria probe Mitotracker Deep Red and with lysosome marker LAMP2 as well (**Figure 5B**), confirming the existence of VDAC1-Rab7a interaction at mitochondrial-lysosome contact sites. In line with the increased VDAC1-Rab7a interaction in *Vam6^+/-^* iNKT cells, these cells displayed elevated mitochondrial-lysosome contacts (**Supplementary Figure 4A**). Meanwhile, the expression levels of mitochondrial maker VDAC1 (**Supplementary Figure 4B**) and lysosomal markers LAMP2 (**Supplementary Figure 4C**) were not influenced by reduced Vam6 expression in iNKT cells, excluding the possible changes in organelle numbers. Next, we investigated whether VDAC1 at mitochondrial-lysosome contact sites regulated interactions between Vam6, Rab7a, and AMPKγ. For this purpose, we generated *Vdac1^+/-^* and *Vdac1^-/-^* mice (**Supplementary Figure 4D**). Reducing VDAC1 expression in iNKT cells impaired mitochondrial-lysosome interaction without influencing the amounts of these two organelles (**Supplementary Figures 4E-G**). Notably, deleting one allele of *Vdac1* significantly enhanced the Rab7a-AMPKγ PLA puncta numbers (**Figure 5C**), the Rab7a-Vam6 PLA puncta numbers (**Figure 5D**), the Vam6-AMPKγ3 PLA puncta numbers (**Figure 5E**), and the AMPKα phosphorylation (**Figure 5F**), whereas significantly reduced S6^S235/236^ phosphorylation (**Figure 5G**) and IFN-γ production (**Figure 5H**), although to a lesser extent than deleting two alleles (**Figures 5C–H**). Additionally, in *Jα18^-/-^* chimeric mice that received CD45.2*^+^ Vdac1^+/-^* bone marrows mixed with CD45.1*^+^ Vdac1^+/+^* bone marrows, CD45.2^+^ *Vdac1* ^+/-^ iNKT cells produced lower levels of IFN-γ and IL-4 than CD45.1^+^ *Vdac1* ^+/+^ iNKT cells did, in response to α-GC injection (**Supplementary Figures 5A, B**). Consistently, in those B16F10 tumor-bearing *Jα18^-/-^* chimeric mice, α-GC induced less IFN-γ and IL-4 production in intratumoral CD45.2^+^ *Vdac1* ^+/-^ iNKT cells, despite their normal granzyme B production (**Supplementary Figures 5C, D**). These results further prove the essential role of VDAC1 in promoting cytokine production in iNKT cells. Moreover, the increased Rab7a-AMPKγ interaction (**Figure 5I**), AMPK activation (**Figure 5J**), and decreased mTOR activation (**Figure 5K**), IFN-γ production (**Figure 5L**), in *Vdac1^+/-^* iNKT cells were recovered by reducing Vam6 expression. These results demonstrate that mitochondrial protein VDAC1 inhibits AMPK activation *via* interfering with Rab7a-Vam6-AMPK complex formation. Given the inhibitory effects of Vam6 on VDAC1-Rab7a interaction as well as lysosome-mitochondria contact, our study imply that Vam6 relieves the inhibitory effect of VDAC1 on Rab7a-Vam6-AMPK complex formation and AMPK activation.

In line with the increased Vam6 expression in intratumoral iNKT cells, we further confirmed that these cells showed reduced mTORC1 activation (**Supplementary Figure 6A**), increased AMPK activation (**Supplementary Figure 6B**), elevated Rab7a-AMPKγ interaction (**Supplementary Figure 6C**), reduced VDAC1-Rab7a interaction (**Supplementary Figure 6D**), and lower lysosome-mitochondria contact (**Supplementary Figure 6E**) than splenic iNKT cells, after activation.

### Lactic acid from tumor cells increases Vam6 expression in intratumoral iNKT cells

**Figure 6:**
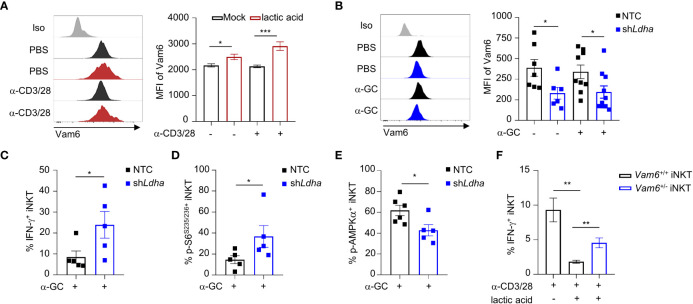
Lactic acid from tumor cells increases Vam6 expression and leads to dysfunction of intratumoral iNKT cells. **(A)** Vam6 expression in splenic iNKT cells activated with or without plate-coated anti-CD3 plus anti-CD28 for 4 hours, in the presence or absence of 5 mM lactic acid. n = 10 replicates for each group. **(B-E)** Vam6 expression **(B**, n = 6-10 mice for each group**)**, IFN-γ production **(C**, n = 5 mice for each group**)**, phosphorylation of S6^S235/S236^ **(D**, n = 5 mice for each group**)**, and phosphorylation of AMPKα **(E**, n = 5-6 mice for each group**)** in intratumoral iNKT cells from *Ldha* knockdown B16F10 tumors or NTC B16F10 tumors, 4 hours post α-GC injection. NTC, none target control cells transfected with scramble shRNA. **(F)** IFN-γ production in splenic *Vam6* ^+/-^ and *Vam6* ^+/+^ iNKT cells, activated by plate-coated anti-CD3 plus anti-CD28 for 18 hours, in the presence or absence of 10 mM lactic acid. n = 6 replicates for each group. Data are shown as mean ± SEM and were analyzed by two-tailed Mann-Whitney tests. *P < 0.05, **P < 0.01, ***P < 0.001.

Previous study has indicated that lactic acid in tumor microenvironment inhibit mTORC1 activation (4). To investigate whether lactic acid in tumor microenvironment elevated Vam6 expression, we treated iNKT cells with lactic acid *in vitro*, and found that lactic acid increased Vam6 expression in both anti-CD3 plus anti-CD28 stimulated and unstimulated cells (**Figure 6A**). To confirm the effects of lactic acid *in vivo*, we knocked down *Ldha* in B16F10 melanoma cells to inhibit production of lactic acid, and subcutaneously injected these *Ldha* knockdown B16F10 cells and normal B16F10 cells into mice, respectively. We showed that iNKT cells isolated from *Ldha* knockdown B16F10 tumors exhibited lower expression of Vam6 than cells isolated from normal B16F10 tumors, no matter these iNKT cells were activated or not (**Figure 6B**), confirming that lactic acid in tumor microenvironment elevated Vam6 expression. In line with their lower level of Vam6, these intratumoral iNKT cells from *Ldha* knockdown B16F10 tumors showed higher levels of IFN-γ (**Figure 6C**) and S6 phosphorylation (**Figure 6D**) but lower level of AMPKα phosphorylation (**Figure 6E**) than those intratumoral iNKT cells from normal B16F10 tumors, in response to α-GC injection. On the other hand, reducing Vam6 expression in lactic acid-treated iNKT cells partially restored their IFN-γ production *in vitro* (**Figure 6F**). These results demonstrate that lactic acid produced by tumor cells increases Vam6 expression in intratumoral iNKT cells and thus leads to impaired cell function.

### *Vam6^+/−^* iNKT cells exhibits enhanced anti-tumor efficacy

**Figure 7:**
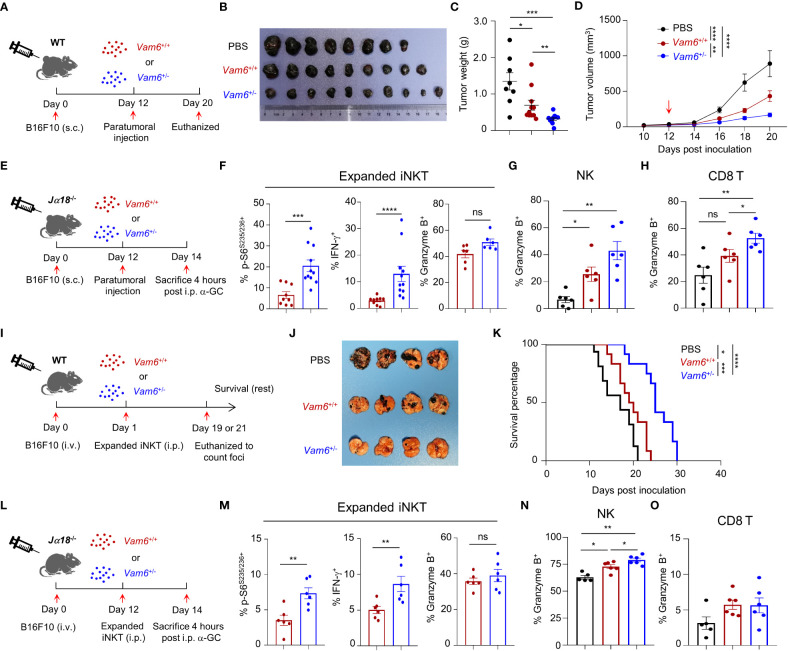
Reduction of Vam6 expression enhances anti-tumor efficacy of expanded iNKT cells. **(A)** Experimental procedure for **(B–D)**. **(B–D)** Representative image of B16F10 tumors **(B)**, tumor weight on day 20 **(C)**, and tumor size **(D)** in wide-type mice receiving PBS, expanded *Vam6* ^+/+^ iNKT cells, and expanded *Vam6* ^+/-^ iNKT cells, respectively. n = 9-10 mice for each group. Red arrow in **(D)** indicates time point of paratumoral injection. **(E)** Experimental procedure for **(F–H)**. **(F–H)** S6^S235/S236^ phosphorylation, IFN-γ production, and granzyme B production in transferred expanded iNKT cells **(F)**, and granzyme B production in NK cells **(G)** and CD8 T cells **(H)** in tumors of *Jα18* ^-/-^ mice received PBS, expanded *Vam6* ^+/+^ iNKT cells, and expanded *Vam6* ^+/-^ iNKT cells, respectively, after α-GC injection on Day 14. n = 6-11 mice for each groups. **(I)** Experimental procedure for **(J, K)**. **(J)** Representative image of lungs with melanoma foci from wide-type mice injected with B16F10 cells (i.v.) and received PBS, expanded *Vam6* ^+/+^ iNKT cells, and expanded *Vam6* ^+/-^ iNKT cells, respectively. n = 7 mice for each group. **(K)** Survival rates of mice injected with B16F10 cells (i.v.) and received PBS, expanded *Vam6* ^+/+^ iNKT cells, and expanded *Vam6* ^+/-^ iNKT cells, respectively. n = 12-16 mice for each group. **(L)** Experimental procedure for **(M–O)**. **(M–O)** S6^S235/S236^ phosphorylation, IFN-γ production, and granzyme B production in transferred expanded iNKT cells **(M)**, and granzyme B production in NK cells **(N)** and CD8 T cells **(O)** in lungs of *Jα18* ^-/-^ mice injected with B16F10 cells (i.v.) and received PBS, expanded *Vam6* ^+/+^ iNKT cells, and expanded *Vam6* ^+/-^ iNKT cells, respectively, after α-GC injection on Day 14. n = 5-6 mice for each groups. Data are shown as mean ± SEM **(C**-**D**, **F–H**, **M–O)** and were analyzed by Two-tailed Mann-Whitney tests **(C**, **F–H, M–O)**, Two-way ANOVA **(D)**, and Log-rank (Mantel-Cox) test **(K)**. *P < 0.05, **P < 0.01, ***P < 0.001, ****P < 0.0001. ns, not significant.

To study the anti-tumor efficacy of *Vam6^+/−^* iNKT *in vivo*, we transferred expanded *Vam6^+/−^* iNKT cell, expanded *Vam6^+/+^* iNKT cells, and PBS into wide-type mice bearing subcutaneous B16F10 tumor cells, respectively (**Figure 7A**). The expanded *Vam6^+/-^* iNKT cells exhibited better anti-tumor efficacy than expanded *Vam6^+/+^* iNKT cells, as indicated by lower tumor weight and smaller tumor size (**Figures 7B-D**). Previous studies have reported that enhancing iNKT cell activation leads to augmented activation of NK and CD8 T cells, both of which could contribute to increased anti-tumor efficacy (5, 22). We found that, upon α-GC injection, expanded *Vam6* ^+/−^ iNKT cells in B16F10 tumors of *Jα18* ^-/-^ mice showed higher level of S6 phosphorylation and IFN-γ production but similar level of granzyme B production, in comparison with expanded *Vam6* ^+/+^ iNKT cells (**Figures 7E, F**). These results imply that expanded *Vam6* ^+/−^ iNKT cells increased tumor clearance *via* indirect manner. Indeed, we found that intratumoral NK cells and CD8 T cells in mice received expanded *Vam6* ^+/−^ iNKT cells produced more granzyme B than those cells in mice received expanded *Vam6* ^+/+^ iNKT cells, indicating a contribution of NK cells and CD8 T cells in enhanced tumor clearance (**Figures 7G, H**). Furthermore, we tested the anti-tumor effect of expanded *Vam6^+/−^* iNKT cells with tumor metastasis models. We respectively transferred expanded *Vam6^+/−^* iNKT cells, expanded *Vam6^+/+^* iNKT cells, and PBS into wide-type mice that were intravenously injected with B16F10 tumor cells one day before (**Figure 7I**). Again, transfer of expanded *Vam6* ^+/-^ iNKT cells better inhibited lung metastasis (**Figure 7J**) and prolonged mouse survival (**Figure 7K**) than transfer of expanded *Vam6* ^+/+^ iNKT cells. Consistently, expanded *Vam6* ^+/−^ iNKT cells in lungs of *Jα18* ^-/-^ metastasis models showed higher level of S6 phosphorylation and IFN-γ production but similar level of granzyme B production in response to α-GC injection, in comparison with expanded *Vam6* ^+/+^ iNKT cells (**Figures 7L, M**). Moreover, we found that NK cells in lungs of mice received expanded *Vam6* ^+/−^ iNKT cells produced more granzyme B than those cells in mice received expanded *Vam6* ^+/+^ iNKT cells, despite similar level of granzyme B in CD8 T cells, indicating a contribution of NK cells in enhanced tumor clearance in lung metastasis models (**Figures 7N, O**). Together, our results demonstrate enhanced anti-tumor efficacy of iNKT cells with reduced Vam6 expression.

## Discussion

In this study, we demonstrate that reduction of Vam6 expression impairs AMPK activation and thus increases mTORC1 activation in iNKT cells. Recruitment of AMPK to lysosome is essential for its activation, and that is mediated through protein interactions between AMPK, AXIN, LKB1, and LAMTOR1 (9). In addition to these proteins, our results indicate that Vam6 plays an essential role in recruiting AMPK to lysosome and activating AMPK *via* promoting formation of Rab7a-Vam6-AMPK complex at lysosome. On the other hand, mitochondrial protein VDAC1 at mitochondria-lysosome contact site interferes with this complex formation *via* protein-protein interaction, and this inhibitory effect of VDAC1 is relieved by Vam6. It is rational that this de-suppressive effect of Vam6 would help to stabilize the Rab7a-Vam6-AMPK complex, in addition to the adaptor protein function of Vam6 in complex formation.

Here, our results reveal that the mitochondria-lysosome contact site serves as a platform for blocking AMPK activation in iNKT cells *via* VDAC1-mediated inhibition of Rab7a-Vam6-AMPK complex formation. Although mitochondria-lysosome contact has been identified for years, the molecules tethering and regulating the contact as well as its contribution to cell fate and functional determination remain unclear. In yeasts, the mitochondria-vacuole contact is mediated by interaction between Vam6, Ypt7 (Rab7a homologue), and Tom40 (23). In contrary to its role in yeasts, Vam6 in iNKT cells serves as a negative regulator that interferes with VDAC1-Rab7a interaction and mitochondria-lysosome interaction. Our data demonstrate an essential role of VDAC1 in tethering mitochondria and lysosome in iNKT cells. Mitochondrial protein VDAC1 has been shown to control the calcium transport at mitochondria-lysosome contact site (24). In addition to favoring the molecule transport between mitochondria and lysosome, our study indicates that VDAC1 also modulates activity of AMPK-mTORC1 pathways at the contact site between these two organelles. In line with our results, lysosomal Rab7a has been previously reported to maintain mitochondria-lysosome contact in both mammalian cells (25) and yeasts (23). A previous study focusing on Rab7a GTPase-activating protein TBC1D15 suggests that active Rab7a promotes mitochondria-lysosome contact (26). Active Rab7a but not inactive Rab7a exhibits lysosomal location (27). Despite the discrepancies in Vam6’s potential guanidine exchange factor (GEF) activity toward Rab7a (28, 29), we found that reduction of Vam6 increased VDAC1-Rab7a interaction at lysosome and promoted mitochondria-lysosome contact. It is rational that Vam6 inhibits the VDAC1-Rab7a interaction and mitochondria-lysosome contact independently of its GEF activity, but through protein-protein interaction.

Our findings that Vam6 promotes AMPK activation and diminishes mTORC1 activation in iNKT cells are in line with a recent study on *Vam6* knockdown myoblasts (12). On the other hand, another study on senescent conventional T cells demonstrates sustained activation of mTORC1 in a lysosomal function independent but Vam6-controlled late endosome dependent manner (13). In the same study, the contribution of Vam6 to mTORC1 activation in young T cells differs at different time windows (13). Additionally, Vam6 in fission yeasts has been reported to activate mTORC1 through activating Gtr1-Gtr2 (30). Whether the senescent T cells and the fission yeasts share similar mechanisms in controlling mTORC1 activation remain to be revealed. It is possible that mTORC1 pathway is regulated by different mechanisms depending on cell type, spatial and temporal distribution of mTOR and Vam6, and other pathways involved.

Notably, iNKT cells are good candidates for anti-tumor immunotherapy (3, 5). Impaired activation of mTORC1 hinders anti-tumor function of intratumoral iNKT cells (4). Here, we demonstrate that lactic acid produced by tumor cells increases Vam6 expression in intratumoral iNKT cells and thus leads to impaired mTORC1 activation and cell function. Although we did not measure the vam6 expression and mTORC1 activation in human intratumoral iNKT cells, published scRNA-seq data of colorectal liver metastases (CRLM) patients from other group (31) show a slightly increase of *Vam6* and impaired mTORC1 activation in intratumoral CD27^+^ iNKT cell cluster, in comparison with cells in para-carcinoma tissues (**Supplementary Figure 7**). These data imply that Vam6-mTORC1 regulatory axis might contribute to the dysfunction of intratumoral iNKT cells in human patients. On the other hand, our findings that reducing Vam6 expression in iNKT leads to enhanced iNKT cell function and exhibits augmented anti-tumor efficacy shed light on future gene editing in iNKT cell-based immunotherapy against tumors.

## Data availability statement

The datasets presented in this study can be found in online repositories. The name of the repository and accession number can be found below: NCBI Sequence Read Archive; PRJNA884874.

## Ethics statement

The animal study was reviewed and approved by Animal Care and Use Committee of the University of Science and technology of China.

## Author contributions

SB, QW, XC, MS, and JP performed experiments, TY and LX generated knockout mice, SZ, YZ, SL, DX, CT, DZ, LW, SF, XL, AJ, DL, JH, YX, TX, ZT, and RZ provided materials, developed methods, or discussed experiments. SB, QW, HZ, and LB conceived the idea, designed the experiments, and wrote the manuscript. All authors contributed to the article and approved the submitted version.
