# Supplementary material for: Vam6 reduces iNKT cell function in tumor via modulating AMPK/mTOR pathways
Source: Front Immunol. 2023 Jan 19;13:1051045. doi: 10.3389/fimmu.2022.1051045 (PMC9892639; doi:10.3389/fimmu.2022.1051045)
Supplement: Supplementary file 1 [file DataSheet_1.pdf]

Supplementary Information for

Vam6 reduces iNKT cell function in tumor via modulating AMPK/mTOR pathways

Supplementary

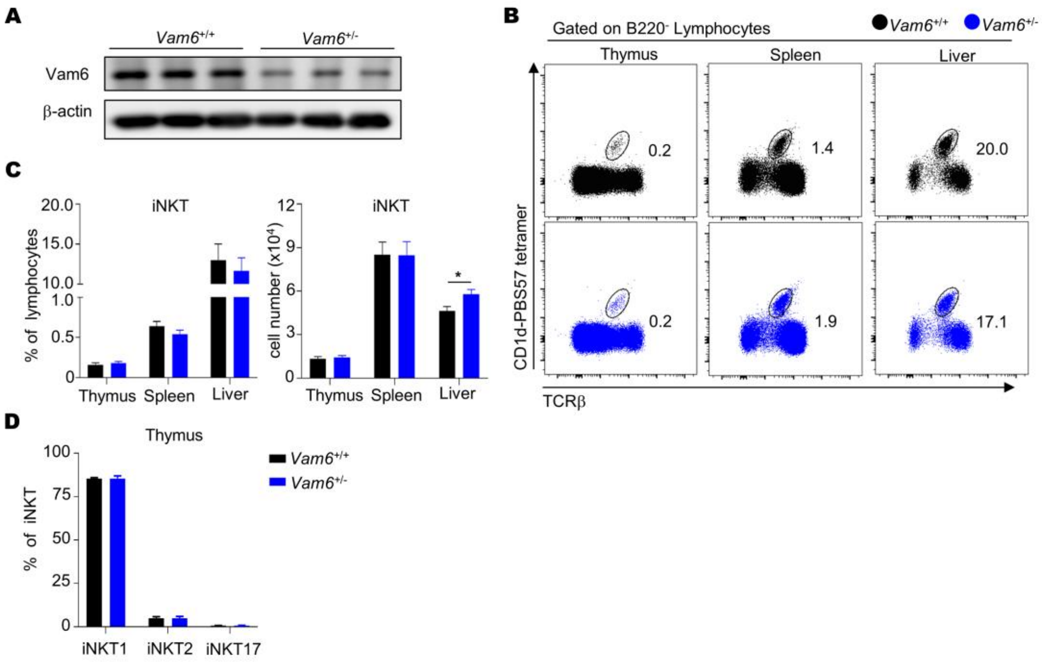

Supplementary Figure 1. Reducing Vam6 expression has no influences on iNKT cell development. **(A)** Expression level of Vam6 in splenic T cells from *Vam6*$^{+/+}$ and *Vam6*$^{+/-}$ mice. n = 3 mice for each group. **(B-D)** Representative flow cytometry dot plots **(B)**, frequencies and absolute numbers **(C)**, and frequencies of subsets **(D)** of iNKT cells in indicated tissues from *Vam6*$^{+/+}$ and *Vam6*$^{+/-}$ mice. n = 5 mice for each group. Data are shown as mean±SEM, pooled from two independent experiments, and analyzed by two-tailed Mann-Whitney tests in **(C)**. *P < 0.05.

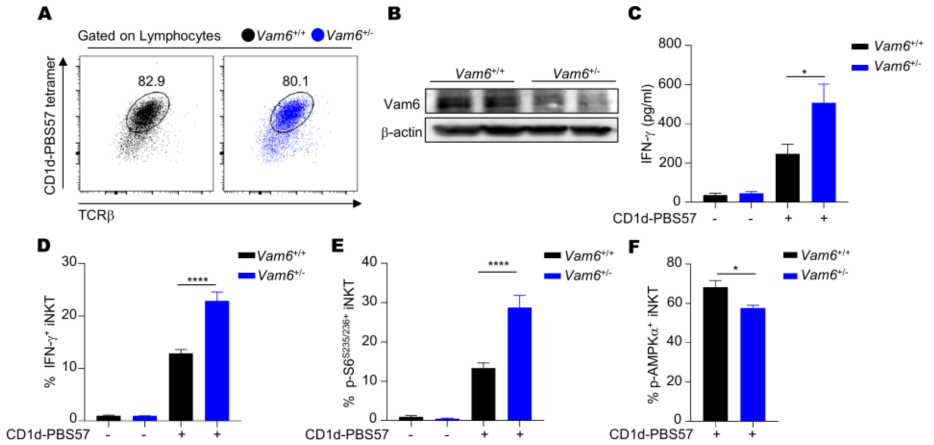

Supplementary Figure 2. Reducing Vam6 expression in expanded iNKT cells influences cytokine production and AMPK/mTORC1 activities. **(A-B)** Purities (**A**) and Vam6 expression level **(B)** in expanded *Vam6*$^{+/+}$ and *Vam6*$^{+/-}$ iNKT cells. **(C-F)** IFN-γ production in supernatants **(C)**, intracellular IFN-γ **(D)**, phosphorylation of S6$^{S235/S236}$ **(E)**, and phosphorylation of AMPK α **(F)** in expanded *Vam6*$^{+/+}$ and *Vam6*$^{+/-}$ iNKT cells stimulated with or without CD1d-PBS57 tetramer. n = 9-10 samples for each group. Data are shown as means ± SEM and pooled from three independent experiments in **(C-F)**. Two-tailed Mann-Whitney test was applied in **(C-F)**. *$P < 0.05$, ****$P < 0.0001$.

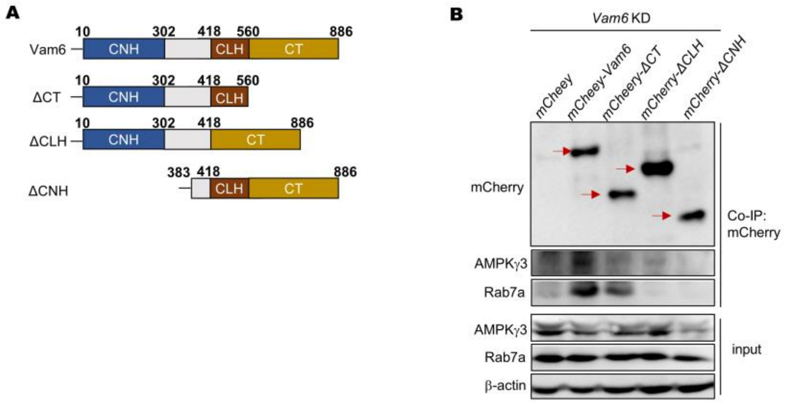

Supplementary Figure 3. Essential region of Vam6 for binding AMPK and Rab7a. **(A)** Diagram showing Vam6 truncations. **(B)** Levels of AMPKγ3 and Rab7a co-immunoprecipitated with mCherry in NIH-3T3 cells expressing *mCherry*, *mCherry-Vam6*, m*cherry*-ΔCT, m*cherry*-ΔCLH, and *mcherry*-ΔCNH, respectively. Red arrows indicate mCherry, mCherry-Vam6, mcherry-ΔCT, mcherry-ΔCLH, and cherry-ΔCNH, respectively.

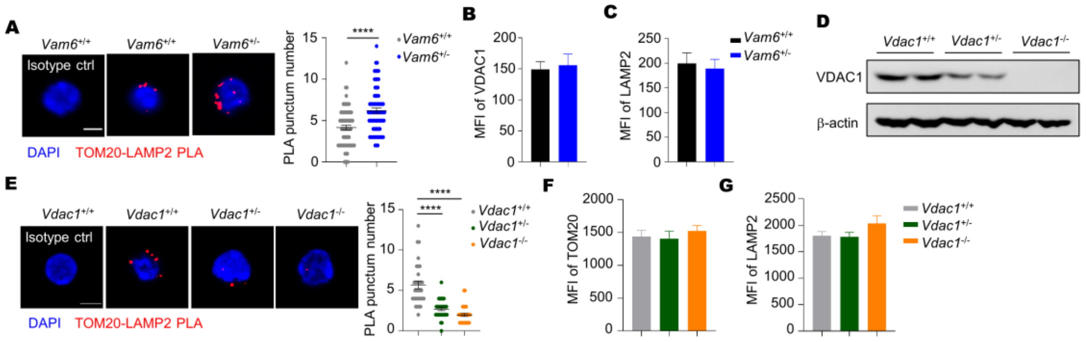

Supplementary Figure 4. Vam6 and VDAC1 control mitochondria-lysosome contact. **(A-C)** TOM20-LAMP2 PLA puncta **(A**, n = 62-66 cells for each group**)**, levels of VDAC1 **(B**, n = 9 replicates for each group**)**, and levels of LAMP2 **(C**, n = 9 replicates for each group) in *Vam6*$^{+/+}$ and *Vam6*$^{+/-}$ iNKT cells stimulated with anti-CD3 plus anti-CD28 for 4 hours. Scale bar, 3 µm. **(D)** Expression level of VDAC1 in splenic T cells from *Vdac1*$^{+/+}$, *Vdac1*$^{+/-}$, and *Vdac1*$^{-/-}$ mice. n = 2 mice for each group. **(E-G)** TOM20-LAMP2 PLA puncta (**E**, n = 32-33 cells for each group), levels of Tom20 **(F**, n = 12 replicates for each group**)**, and levels of LAMP2 **(G**, n = 12 replicates for each group**)** in *Vdac1*$^{+/+}$, *Vdac1*$^{+/-}$, and *Vdac1*$^{-/-}$ iNKT cells stimulated with CD1d-PBS57 tetramer for 4 hours. Data are shown as mean ± SEM and pooled from three independent experiments in **(A-C)** and **(E-G)**. Data were analyzed by two-tailed unpaired Student's t test (**A** and **E**) and two-tailed Mann-Whitney tests **(B-C** and **F-G)**. ****P < 0.0001.

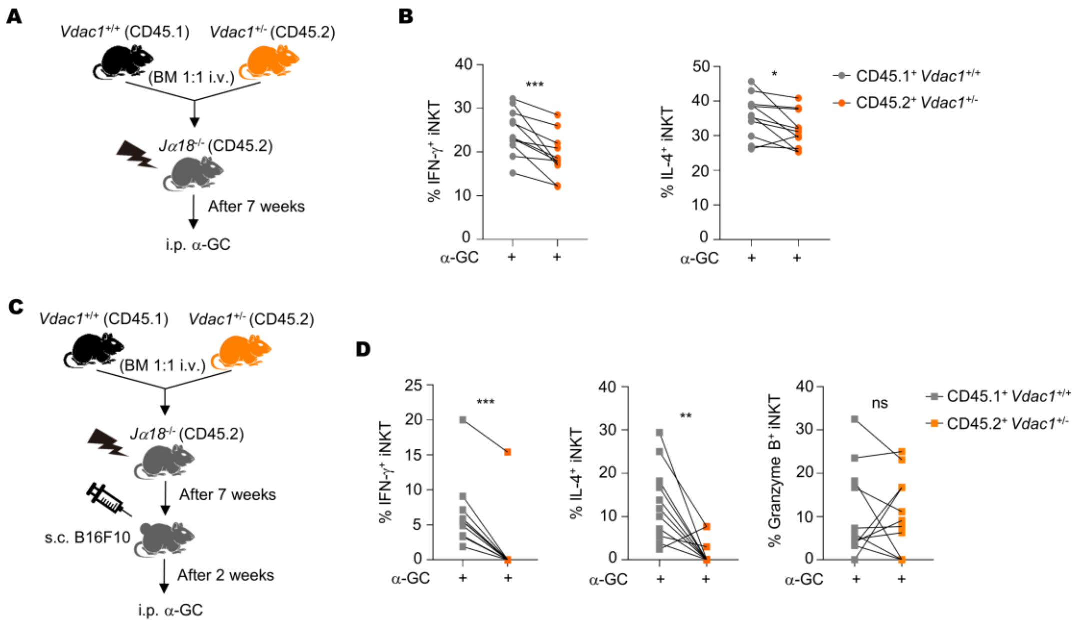

Supplementary Figure 5. VDAC1 inhibits cytokine production in iNKT cells. **(A)** Experimental procedure for **(B)**. **(B)** IFN-γ and IL-4 production in CD45.1^{+} *Vdac1*$^{+/+}$ iNKT cells and CD45.2^{+} *Vdac1*$^{+/-}$ iNKT cells in spleens of chimeric mice, 4 hours after α-GC or PBS injection. n = 11 mice. **(C)** Experimental procedure for **(D)**. **(D)** IFN-γ, IL-4, and granzyme B production in CD45.1^{+} *Vdac1*$^{+/+}$ iNKT cells and CD45.2^{+} *Vdac1*$^{+/-}$ iNKT cells in B16F10 tumors of chimeric mice, 4 hours post α-GC injection. n = 11 mice. Data were analyzed by two-tailed Wilcoxon matched-pairs signed rank tests. *P < 0.05, **P < 0.01, ***P < 0.001. ns, not significant.

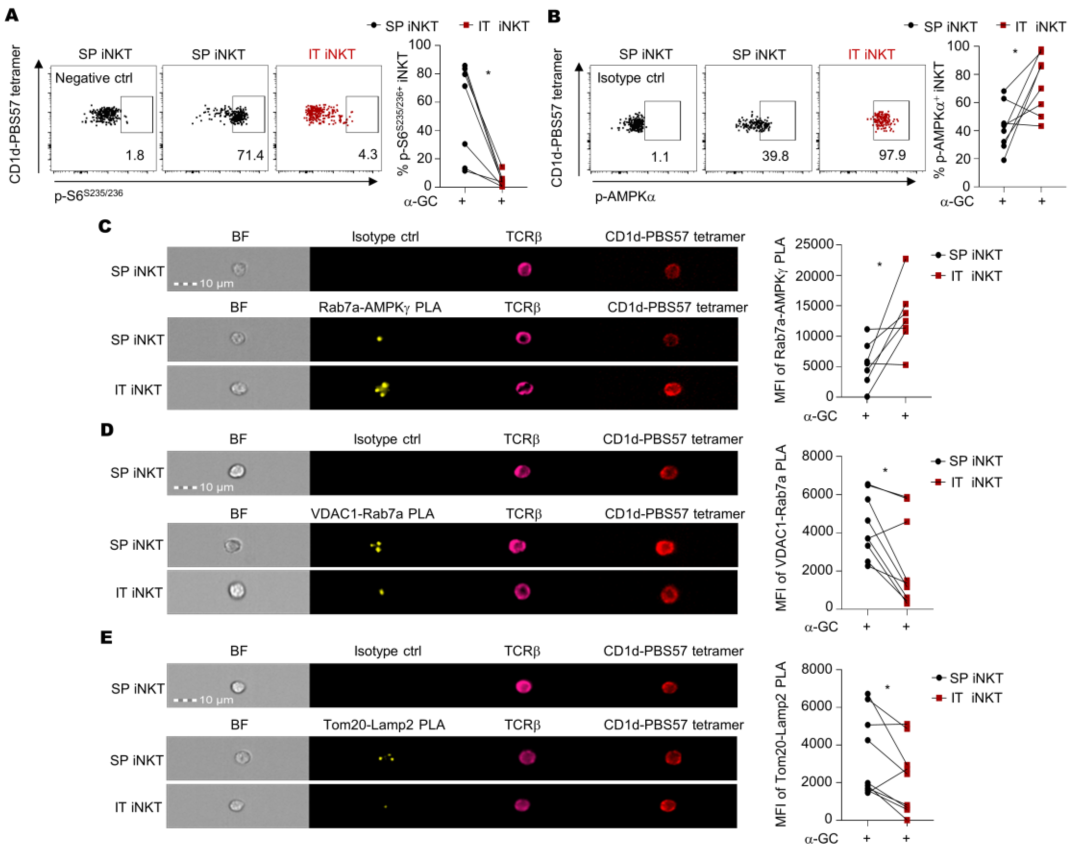

Supplementary Figure 6. Intratumoral iNKT cells show lower mTORC1 activation and VDAC1-Rab7a interaction but higher AMPK activation and Rab7a-AMPKγ interaction. **(A-B)** Phosphorylation of $S6^{S235/S236}$ **(A**, n = 7 mice**)**, and phosphorylation of AMPKα **(B**, n = 8 mice**)** in splenic (SP) and intratumoral (IT) iNKT cells from MC38 tumor-bearing mice, 4 hours post α-GC injection. **(C-E)** Median fluorescence intensity (MFI) of Rab7a-AMPKγ PLA **(C**, n = 7 mice**)**, MFI of VDAC1-Rab7a PLA **(D**, n = 9 mice**)**, and MFI of TOM20-LAMP2 PLA **(E**, n = 9 mice**)** detected by image flow cytometry in splenic (SP) and intratumoral (IT) iNKT cells from MC38 tumor-bearing mice, 4 hours post α-GC injection. Data are pooled from three **(B** and **E)**, or four **(C-D)** independent experiments and were analyzed by two-tailed Wilcoxon matched-pairs signed rank tests. $^*P < 0.05$.

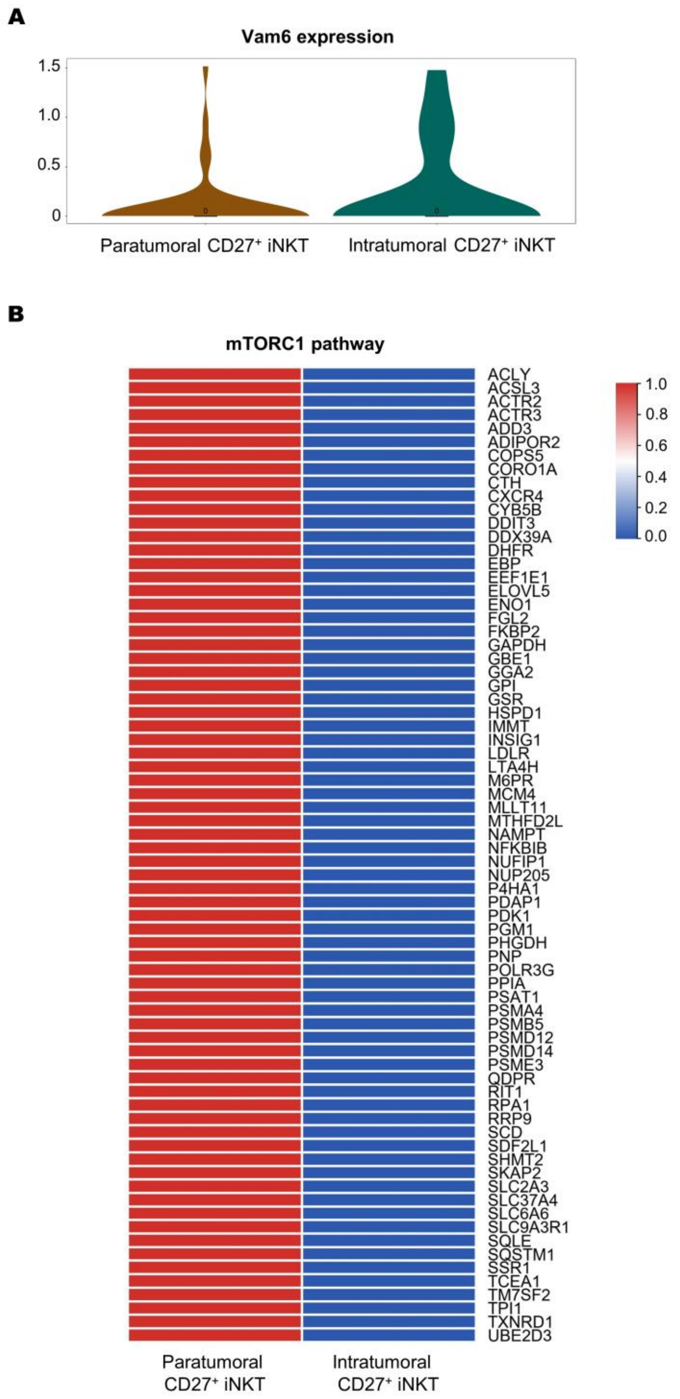

Supplementary Figure 7. Published scRNA-seq data show impaired Vam6-mTORC1 axis in $CD27^+$ iNKT cells in tumors of CRLM patients. **(A-B)** Vam6 expression **(A)** and heatmap of mTORC1 pathway related genes **(B)** in $CD27^+$ iNKT cells from para-carcinoma tissues and primary tumors of CRLM patients (GEO: GSE164522).
